# Adrenal cortical carcinoma masquerading as pheochromocytoma: a case report

**DOI:** 10.3332/ecancer.2012.277

**Published:** 2012-10-31

**Authors:** H Ni, A Htet

**Affiliations:** 1 Department of Medicine, Melaka Manipal Medical College, Melaka 75150, Malaysia; 2 Department of Diagnostic Radiology, Naypyitaw Defence Service General Hospital, Naypyitaw, Myanmar

**Keywords:** adrenocortical carcinoma, pheochromocytoma, adrenal tumours, paroxysmal hypertension, urinary vanillyl mandelic acid

## Abstract

Adrenocortical carcinoma (ACC) is a malignant tumour arising from the adrenal cortex, while pheochromocytoma is a catecholamine secreting tumour of the adrenal medulla or extra adrenal sites. Both conditions are very rare, with incidence of approximately 1–2 cases per million adults annually. Most adrenocortical tumours are functioning. ACC can be associated with clinical Cushing syndrome and virilisation due to excessive production of cortisol and androgens, respectively. However, it is rare for ACC to present clinically as pheochromocytoma. We report a case of a 28-year-old lady who presented with paroxysmal hypertension and palpitations associated with raised urinary vanillyl mandelic acid. On examination, there was postural hypotension and ballotable mass in right lumbar region with no obvious features suggestive of Cushing syndrome or virilisation. A huge right suprarenal mass with areas of necrosis and calcification was noted on the abdomen CT. A right adrenalectomy was done. The histology was consistent with ACC. There are reported cases of ACC presenting with clinical features of pheochromocytoma but limited in number, accounting for barely a dozen cases in the literature. This pseudopheochromocytoma may be due to the presence of neuroendocrine features in ACC.

## List of abbreviations

BP: Blood pressure
CT: Computed tomography
CXR: Chest X-ray
ECG: Electrocardiogram
MRI: Magnetic resonance imaging
VMA: Vanillyl mandelic acid
ACC: Adrenocortical carcinoma
PET: Positron emission tomography

## Introduction

Though benign adreno-cortical tumours are common, adrenocortical carcinoma (ACC) is a relatively rare condition with diverse clinical manifestations due to excessive hormone production by the tumour, with Cushing syndrome and virilisation being the commonest clinical features [1]. Pheochromocytoma is the catecholamine-secreting paragangliomas arising from adrenal or extra-adrenal sites [2]. There are reported cases of concurrence of pheochromocytoma and ACC [1]. However, it is rare for ACC to present clinically as pheochromocytoma. In this case, we report a case of ACC mimicking pheochromocytoma clinically and biochemically.

## Clinical case

A 28-year-old single lady presented with palpitations on and off for one and half years, which was episodic with no obvious specific precipitating factors. These attacks were accompanied by excessive sweating, flushing, and cold extremities. She also experienced dizziness during change of posture particularly standing up from sitting. However, she denied fear of impending death or syncopal attacks. She was also diagnosed with hypertension one and a half years back at her first presentation of palpitation. She took prescribed antihypertensive medications irregularly. Her highest BP was 220/110 mmHg, and sometimes BP was stable without any medication. She noticed some degree of weight loss during these years.

On physical examination, there were no features of virilisation or Cushing syndrome. Pulse rate was 120, regular with no special character or radiofemoral delay. Postural hypotension was noted with lying BP 140/100 mmHg and standing BP 120/90 mmHg. Precordium and respiratory examination were normal. There was a mass palpable in the right lumbar region, 5 × 5 cm, firm in consistency and ballotable. No tenderness or renal bruit noted. Left kidney was not ballotable. Grade II hypertensive retinopathy was seen on fundoscopy.

**Figure 1:**
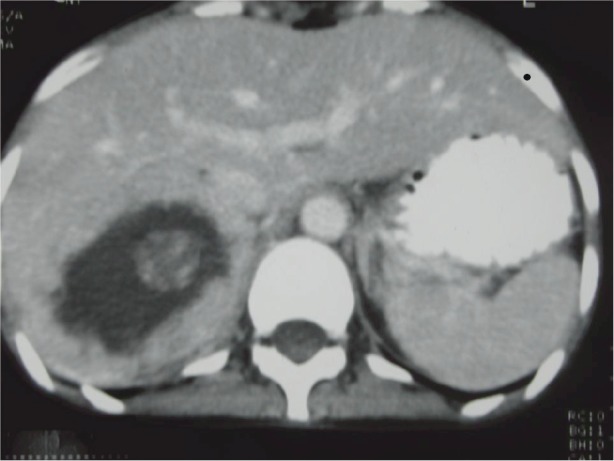
CT abdomen. Huge right suprarenal mass measuring 16.5 × 6.5 × 8.7 cm.

**Figure 2:**
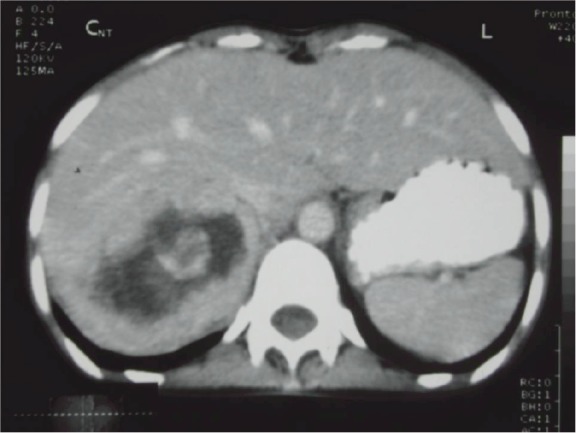
CT abdomen. Right adrenal mass with break down areas and calcifications.

Her ECG showed T wave inversion in leads I, aVL, V4-V6 with no evidence of left ventricular hypertrophy. CXR and serum electrolytes were normal. Ultrasonography of abdomen revealed right supra-renal mass of mixed echogenicity measuring 13.1 cm × 7.7 cm. On CT abdomen, there was a huge well-circumscribed soft tissue mass of 16.5 × 6.5 × 8.7 cm in right retroperitoneal region with break down areas and calcifications (Figures 1 and 2). A marked contrast enhancement in this right retroperitoneal mass was evident in post-contrast films, giving the impression of right adrenal malignant tumour. Serum cortisol was within normal limits, however, urinary VMA level was 21 mg/24 h, three times higher (normal: 1.8–7.1 mg/24 h). Due to limited resources, further imaging studies such as indium-111-octreotide or gallium 68-DOTA (tetraazacyclododecane tetraacetic acid) positron emission tomography (PET) to help localize other possible sources of catecholamine secreting neuroendocrine tumours could not be done.

The operation was performed by the urosurgical team after stabilization, which revealed a tumour mass of 17.8 × 12.7 × 7.6 cm in the right adrenal gland with adhesions to the right lobe of the liver and peritoneal wall. A right adrenalectomy was done. The histopathology reported an encapsulated adrenal tumour arising from the cortex, predominantly yellow on the cut surface with areas of haemorrhage, calcification, and necrosis. A moderate degree of atypia was noted, consisting of giant cells and spindle cells. However, there was no invasion into the adrenal vein or lymphatics. Because of limited resources, immunohistochemical staining cannot be performed. The impression of biopsy result was adreno-cortical carcinoma.

Recheck urinary VMA 2 weeks after surgery was 5.4 mg/24 h, which was normal. Her BP was controlled with labetalol. The post-operative period was uneventful with improvement of symptoms.

## Discussion

Adrenocortical carcinoma (ACC) is a very rare condition, accounting for 1–2 cases per million per year in adults [1, 2]. Genetic and environmental factors play a role in the pathogenesis [2].

About one quarter to three quarters of ACC is functioning with excess hormonal production [3]. Various types of steroid are produced by ACC. Secretion of both cortisol and androgen is most common and is very much suggestive of malignant adrenocortical tumour [2]. Cortisol over secretion causes clinical Cushing syndrome, and excess androgen leads to virilisation in females, both of which can be the first manifestation in ACC [1].

Pheochromocytoma is a catecholamine secreting tumour which arises from adrenal medulla. It is also a rare neoplasm, with an estimated incidence of 2–8 cases per million per year [4]. It accounts for 0.1% of secondary hypertension cases [1]. It can be sporadic or associated with familial syndromes, such as multiple endocrine neoplasia (MEN) 2A and 2B, von Hippel–Lindau (VHL) disease, and neurofibromatosis type 1 [5]. Catecholamine excess in pheochromocytoma causes sustained or episodic hypertension associated with paroxysmal symptoms or “spells” of palpitations, sweating, headache, and panic attacks [6]. Depending on the catecholamines produced, hypertension in pheochromocytoma is paroxysmal in 48%, persistent in 29%, whereas 13% have normal blood pressure. Norepinephrine-secreting tumours cause sustained hypertension, while tumours that secrete large amounts of both epinephrine and norepinephrine are associated with episodic hypertension. In contrast, tumours producing epinephrine alone cause hypotension rather than hypertension [7]. In the present case, hypertension was episodic; however, plasma catecholamine levels were not available. PET imaging using Gallium 68 DOTA compounds has been shown to improve the quality of imaging of neuroendocrine tumours including pheochromocytoma [8].

The size and appearance on imaging studies such as CT or MRI (magnetic resonance imaging) are useful in distinguishing benign adrenal tumours from ACC. The mean tumour size of ACC at the diagnosis was 11.5 ± 4.7 cm (range 3–28 cm) [9]. The size of the adrenal mass in our patient was 16.5 cm. Other imaging features suggestive of ACC include irregular margin, inhomogeneous appearance, presence of necrotic areas, calcifications, low-fat content, high attenuation on unenhanced CT with irregular enhancement after contrast [9, 10]. In the present case, there were areas of calcification and necrosis on CT together with marked contrast enhancement, indicating the malignant nature of right adrenal tumour. Intra-operative findings of adhesion to liver and peritoneal membrane further support malignancy of adrenal gland in this patient.

Pathologically malignant adrenal tumours are differentiated from benign lesions by gross appearance of tumour weight, haemorrhages, capsular involvement as well as microscopic diagnostic score of Weiss. Moreover, broad fibrous bands, nuclear atypia, and necrosis are suggestive of malignancy [9]. The presence of at least one of the parameters: necrosis, high-mitotic rate, and venous or capsular invasion further contribute to the diagnosis of ACC [11]. Nuclear atypia and necrosis were present in our patient but no vascular invasion was noted. Immunohistochemical staining is positive for neurone-specific enolase and chromogranin in pheochromocytomas whereas inhibin for adrenocortical neoplasms. Testing for calretinin in addition to inhibin further increases the sensitivity and specificity in differentiation of adrenocortical neoplasm from pheochromocytoma [12].

It is extremely uncommon for ACC to present as pheochromocytoma. The initial presentation combined with biochemical evidence of excessive urinary VMA level lead to preoperative diagnosis of pheochromocytoma in our case. In a case report dated back to 1962, a 44-year-old man presented clinically with paroxysmal hypertension and sweating with raised catecholamines but histology revealed ACC [13]. Another report featured two patients with ACC presenting with clinical findings similar to pheochromocytoma (the so-called pseudopheochromocytoma). These clinical findings are explained by the presence of neuroendocrine features in these adrenal tumours [14]. In the literature, there are reported cases where both adrenocortical tumours and pheochromocytoma coexist as well [1, 15].

In the present case, it was not the concurrence of both tumours but the functioning carcinoma of the adrenal cortex presenting with clinical features of pheochromocytoma which is unique and rare. To the best of our knowledge, this is the first reported case of ACC presenting with pheochromocytoma features from Myanmar.
